# Accuracy of Heart Rate Measurement by the Fitbit Charge 2 During Wheelchair Activities in People With Spinal Cord Injury: Instrument Validation Study

**DOI:** 10.2196/27637

**Published:** 2022-01-19

**Authors:** Dirk Hoevenaars, Iris E Yocarini, Stylianos Paraschiakos, Jasmijn F M Holla, Sonja de Groot, Wessel Kraaij, Thomas W J Janssen

**Affiliations:** 1 Faculty of Behavioural and Movement Sciences Vrije Universiteit Amsterdam Amsterdam Netherlands; 2 Amsterdam Rehabilitation Research Center Reade Amsterdam Netherlands; 3 Leiden Institute of Advanced Computer Science Leiden University Leiden Netherlands; 4 Molecular Epidemiology Department of Biomedical Data Science Leiden University Medical Center Leiden Netherlands; 5 Faculty of Health, Sports and Social Work Inholland University of Applied Sciences Haarlem Netherlands; 6 Center for Adapted Sports Amsterdam Institute of Sport Science Amsterdam Netherlands

**Keywords:** Fitbit Charge 2, heart rate, accuracy, photoplethysmography, spinal cord injury

## Abstract

**Background:**

Heart rate (HR) is an important and commonly measured physiological parameter in wearables. HR is often measured at the wrist with the photoplethysmography (PPG) technique, which determines HR based on blood volume changes, and is therefore influenced by blood pressure. In individuals with spinal cord injury (SCI), blood pressure control is often altered and could therefore influence HR accuracy measured by the PPG technique.

**Objective:**

The objective of this study is to investigate the HR accuracy measured with the PPG technique with a Fitbit Charge 2 (Fitbit Inc) in wheelchair users with SCI, how the activity intensity affects the HR accuracy, and whether this HR accuracy is affected by lesion level.

**Methods:**

The HR of participants with (38/48, 79%) and without (10/48, 21%) SCI was measured during 11 wheelchair activities and a 30-minute strength exercise block. In addition, a 5-minute seated rest period was measured in people with SCI. HR was measured with a Fitbit Charge 2, which was compared with the HR measured by a Polar H7 HR monitor used as a reference device. Participants were grouped into 4 groups—the no SCI group and based on lesion level into the <T5 (midthoracic and lower) group, T5-T1 (high-thoracic) group, and >T1 (cervical) group. Mean absolute percentage error (MAPE) and concordance correlation coefficient were determined for each group for each activity type, that is, rest, wheelchair activities, and strength exercise.

**Results:**

With an overall MAPE_all lesions_ of 12.99%, the accuracy fell below the standard acceptable MAPE of –10% to +10% with a moderate agreement (concordance correlation coefficient=0.577). The HR accuracy of Fitbit Charge 2 seems to be reduced in those with cervical lesion level in all activities (MAPE_no SCI_=8.09%; MAPE_<T5_=11.16%; MAPE_T1−T5_=10.5%; and MAPE_>T1_=20.43%). The accuracy of the Fitbit Charge 2 decreased with increasing intensity in all lesions (MAPE_rest_=6.5%, MAPE_activity_=12.97%, and MAPE_strength_=14.2%).

**Conclusions:**

HR measured with the PPG technique showed lower accuracy in people with SCI than in those without SCI. The accuracy was just above the acceptable level in people with paraplegia, whereas in people with tetraplegia, a worse accuracy was found. The accuracy seemed to worsen with increasing intensities. Therefore, high-intensity HR data, especially in people with cervical lesions, should be used with caution.

## Introduction

### Background

Spinal cord injury (SCI) is a result of a partial or complete disruption of the neuropathways in the spinal cord, causing loss of motor and sensory function and a disturbed autonomic nervous system (ANS). Wheelchair users with SCI have one of the lowest daily activity levels compared with other groups with chronic physical conditions [1], negatively affecting their daily activity energy expenditure. In addition, their resting energy expenditure is often decreased because of multiple factors, with a reduced fat-free mass as a major contributor [2-5]. Together with the reduced activity energy expenditure, this leads to a lower total daily energy expenditure. As a consequence, approximately 68% of the people with SCI are overweight or obese, associated with increased risks of cardiovascular disease and mortality [6,7]. Therefore, maintaining or achieving an active lifestyle is even more crucial in people with SCI than in the able-bodied population. There are several tools that can help to stimulate or maintain an active lifestyle. Currently, activity trackers are a popular way to get insight on and monitor one’s personal activity level. Activity trackers include many features, such as estimations of activity levels, exercise intensity or daily energy expenditure, often based on recorded movement via accelerometry and heart rate (HR).

HR is one of the most important and often used physiological parameters, as it is directly related to oxygen consumption and energy expenditure. The delivery of oxygen-rich blood required in the circulation system is controlled by the ANS by modulating both the HR and stroke volume [8,9]. For this reason, HR is used to monitor exercise intensity or as a derivative to estimate, for example, maximal oxygen uptake (VO_2_max), or energy expenditure [10]. Over the last 4 decades, HR during exercise has mainly been measured using HR monitors that make use of a chest belt, transmitter, and receiver. Owing to the rapid development of sensor technology in recent decades, it is now possible to record and track HR in an even less invasive and easier way. One of the most popular and commonly used methods to determine HR in daily life is photoplethysmography (PPG), a simple and low-cost technique that can be integrated in a wrist-worn activity tracker [11,12].

PPG is a technique in which blood volume changes are detected in the microvascular bed of tissue by infrared light reflected from the tissue, such as the ear lobe, finger, or wrist [11]. The change in blood volume after a heartbeat is proportional to the reflected light, allowing pulse wave detection in the wrist, which can be used as a derivative to determine HR [13]. HR recording with this technique, however, is more susceptible to motion artifacts caused by hand-arm movements and blood flow dynamics and can, therefore, lead to a lower accuracy [14,15]. Studies have shown acceptable validity and accuracy (<10%) in HR recordings during sleep or across a 24-hour period in a free-living environment in able-bodied individuals with a mean absolute percentage error (MAPE) of <10% [16,17]. However, when tested during activities of higher intensities or dynamic situations, the accuracy dropped (MAPE>10%) [18-20]. Owing to the developments in HR recording with activity trackers, they are being included in clinical settings for medical purposes, such as mobile health monitoring, noninvasive medical surveillance, or even detecting first signs of health issues [21-23]. As information gathered by activity trackers is more often used for clinical and health purposes, the importance of accurate data is growing. However, as measurement techniques rely on physiological properties and responses, measurement outcomes can differ if physiological responses are altered, for instance, because of medical conditions. Therefore, it is important to investigate the accuracy of HR measurement within different populations, such as in people with SCI, as their physiological responses can be severely altered [24].

### Objectives

The accuracy of HR determined by PPG depends on blood pressure changes which is, among other things, influenced by HR variability [25]. Both, the blood pressure of the upper limbs and HR are regulated by the ANS, of which the sympathetic outflow occurs between the first thoracic (T1) spinal cord segment and the fifth thoracic (T5) spinal segment. After an SCI, neural signal transmission is partially or fully lost at and below the lesion level. In case of an SCI at or above the T5 spinal cord segment, neural signaling and, therefore, the balance between the parasympathetic and sympathetic systems are often altered. Sympathetic hypoactivity usually occurs, resulting in possible low HR, low resting blood pressure, disturbed vascular regulation, and altered responses in these systems during rest or during physical activities [24]. Owing to the changes in HR response and blood pressure control, the accuracy of HR determined by PPG could be affected when a lesion occurs above T5. Because of possible impaired or altered vascular regulation, artifact-reducing algorithms may not apply and might subsequently compromise HR accuracy. The ANS is even more affected in cervical lesions, as the imbalance between the parasympathetic and sympathetic systems increases with lesion level [26]. Therefore, the aim of this study is to evaluate whether Fitbit Charge 2 can accurately record HR in wheelchair users with SCIs and to investigate how lesion level affects accuracy. In addition, the effect of intensity on accuracy is determined during wheelchair activities and strength exercise, as a higher intensity is expected during strength exercise compared with wheelchair activities and during wheelchair activities compared with rest. It is hypothesized that the HR accuracy of the Fitbit Charge 2 is lower in people with lesions at or above T5 because of the possible affected ANS, compared with people with lesions below T5 or without SCI. A further reduction in accuracy is expected in people with a cervical lesion compared with those with a lower lesion level or without SCI, because of an enlarged imbalance between the parasympathetic and sympathetic systems. Furthermore, the accuracy is expected to decrease with increasing intensities.

## Methods

### Study Design

Data on body composition and energy expenditure in people with SCI were collected in a larger cross-sectional study. All participants were invited for a one-time visit to the Amsterdam Nutritional Assessment Center laboratory of the Amsterdam University of Applied Sciences. HR of the participants was recorded during rest, wheelchair activities, and a 30-minute strength exercise block with both the Fitbit Charge 2 and Polar H7 HR monitor. All participants provided signed informed consent before participating. The study was approved by the medical ethical committee of Slotervaart Ziekenhuis—Reade (METc nr. P1805).

### Participants

Overall, 48 participants were recruited to participate in this study, 38 (79%) with SCI and 10 (21%) without SCI. Recruitment took place through advertisements via the Dutch SCI patient association, social media, rehabilitation center Reade in Amsterdam, and the social network of the involved researchers. Participants were included if the following inclusion criteria were met: age between 18 and 75 years; chronic SCI (time since injury >1 year), not ventilator-dependent; and wheelchair-dependent for longer distances. Exclusion criteria were as follows: presence of a pacemaker, severe edema, progressive illness, pressure ulcers, metabolic diseases, severe comorbidities, psychiatric disorders, pregnancy, and insufficient understanding of the Dutch language to understand the study. Participants without SCI were selected based on the same inclusion and exclusion criteria, except for the SCI-related criteria. Personal and lesion characteristics were obtained through a questionnaire and interview. A conservative sample size target was chosen and set on ≥40 samples of each device for each group for each activity based on the method comparison guideline [27].

The participants were divided into 4 groups—the without SCI group and based on their lesion level they were divided into the cervical (>T1), high-thoracic (T1-T5), and midthoracic and lower (<T5) groups, to test the influence of lesion level on PPG accuracy. Heart and upper-body blood vessels are sympathetically innervated from segments T1-T5 and interact with the parasympathetic system to provide a balanced regulation of the cardiovascular system. In people with an SCI at T5 and above, sympathetic innervation is likely to be affected to a certain extent, which causes altered HR response and blood pressure regulation, possibly affecting PPG recordings compared with lower lesions. In addition, the lesion groups T5 and above were divided into the following lesion subgroups: lesion above T1 and lesion between T1-T5, with a larger imbalance in the ANS expected in the first group and thus a more severe cardiovascular dysfunction [28]. In people with an SCI above T1, arm function might be impaired, as well as a more severed impaired sympathetic innervation of the heart and upper-body vessels compared to lower lesions, which could lead to a lower HR accuracy in those with a cervical lesion [29].

### Materials

#### Fitbit Charge 2

The Fitbit Charge 2 (2017 version, Firmware version 22.55.2, Fitbit Inc) is a commercially available activity tracker with multiple sensors, such as a 3-axis accelerometer, an altimeter, and a PPG sensor to record HR. In the Fitbit Charge 2 PurePulse, HR technology is used as an investigational device, which constantly reads the changes in the blood volume at the wrist. An algorithm converts these data into continuous HR data. The smartwatch was tightly positioned according to instructions on Fitbit on the wrist of participants on which normally a watch would be worn, usually the nondominant side. Intraday data collection was requested and approved by Fitbit for research purposes, allowing us to obtain the data on the highest possible sampling rate for the time period in which all activities were performed through an application programing interface. Output frequency of the HR data varied between 0.2 Hz and 0.06 Hz. Data collected by the Fitbit were transferred through Bluetooth Low Energy to the Fitbit App and downloaded.

#### Polar H7 HR Monitor

The Polar H7 chest strap HR monitor (Bluetooth Low Energy version, Polar Electro) was used as a reference device to measure HR; it is an accurate (intraclass correlation coefficient=0.98) alternative for a 3-lead electrocardiography (ECG), which is considered as the gold standard for measuring HR [30]. The strap was moistened to improve conduction between the skin and the sensor before it was secured tightly around the chest. HR recording was connected with a Cortex Metamax 3B (Cortex Biophysik GmbH) portable indirect calorimetry system, used in the larger study, which collects data at each full breathing cycle. Therefore, the output frequency of the Polar H7 HR data was determined by the breathing frequency of the participants during the protocol. The HR output given after each breathing cycle was the average HR measured over the entire breathing cycle.

### Measurement Protocol

After ensuring that all sensors were positioned correctly, the measurement protocol started with a 5-minute seated rest, followed by wheelchair activities, consisting of eleven different wheelchair tasks executed for 1 minute, namely: (1) wheelchair propulsion on a low-resistance surface on a slow, (2) normal, and (3) high speed; (4) handcycling on an armcrank ergometer; (5) rummaging in a bag while being pushed; (6) setting the table; (7) doing dishes; (8) typing on a laptop; (9) maneuvering the wheelchair; (10) wheelchair basketball; and (11) transfer from wheelchair to chair and back. No 5-minute seated rest data were available for the participants without SCI, as this was added to the measurement protocol after finishing the measurements of the participants without SCI. All tasks were performed for 1 minute, as this represents real-life situations better compared with longer steady-state situations. All tasks were timed, logged, and recorded using a camera. Between each task, a rest period allowed the HR to recover close to the resting level to ensure variability in measured HR between tasks. If the participant was not able to perform a wheelchair activity independently because of their impairment, the task was not executed. After the activities were completed, a 30-minute upper-body strength exercise was performed. Exercises and resistances were chosen based on the participants’ preferences and physical capabilities. All strength exercises were performed with sets of 8-12 repetitions, and each set was repeated 3 times in total. After each set, there was a rest period that lasted between 90 and 120 seconds before the next set was started. The strength exercise block was not executed if the participant was not able to perform strength exercises because of an upper-body injury or impairment.

### Data Analyses

#### Missing Data and Synchronization

On the basis of expert evaluation, all data of 8% (4/48) individuals were excluded. Of the 4 individuals, data for 2 (50%) individuals were excluded because of poor Polar H7 HR monitor connection throughout the whole measurement, data for 1 (25%) were excluded owing to battery failure of the Polar H7 HR monitor, and data for 1 (25%) were excluded because of the loss of Fitbit Charge 2 data. In total, the HR data of 92% (44/48) of participants were analyzed. In addition, approximately 0.6% of the data were excluded from 13% (6/48) of participants because of invalid samples (temporary loss of Polar H7 HR monitor connection). In total, 21,732 valid HR samples from both devices were used for analysis. The data of the 2 devices with different sampling rates were synchronized by relating the HR monitored by the reference device (ie, Polar H7 HR monitor) to that of the investigational device (ie, Fitbit Charge 2) that was closest in time. Consequently, data were labeled with one of the three activity categories: rest, wheelchair activities (including resting time between the activities and before the strength exercises started), and strength exercises (including resting time between the exercises) based on logbook data and video recordings.

#### Statistical Analyses

All statistical analyses were performed in R (version 3.6.1; R Foundation for Statistical Computing) using R Studio (version 1.2.1335). To assess error, the mean difference between the Polar H7 HR monitor and Fitbit Charge 2 HR samples was calculated, resulting in the mean error. In addition, the mean absolute error (MAE) and the MAPE were evaluated. As stated by the American National Standards Institute, the accuracy of HR monitors should be within –10% to +10% of the input rate or –5 to +5 beats per minute (bpm), whichever is greater [31]. In alignment with these standards, we considered a MAPE of –10% to +10% as an acceptable error rate. Following Nelson and Allen [17], outliers were not removed to evaluate the accuracy of consumer use conditions. Bland-Altman plots with 95% limits of agreement (LoA) were produced using the BlandAltmanLeh R package [32]. The Bland-Altman plots and LoA are the suggested methods for analyzing the agreement between 2 measurement devices [33-36]. These plots were inspected to assess systematic biases over the entire HR range and to assess the magnitude of such biases and whether Fitbit Charge 2 overestimated or underestimated HR compared with the Polar H7 HR monitor. Finally, in line with previous wearable validation studies [17,33], Lin concordance correlation coefficients (CCCs) [37] were calculated using the DescTools R package [38]. These correlation coefficients provide information on the association and strength of the linear relationships between the reference device and investigational device. According to Nelson and Allen [17], the strength of agreement can be interpreted based on the following: CCC<0.5 indicates a weak association, CCC between 0.5 and 0.7 indicates a moderate association, and CCC>0.7 relates to a strong association.

## Results

### Descriptives

**Figure 1:**
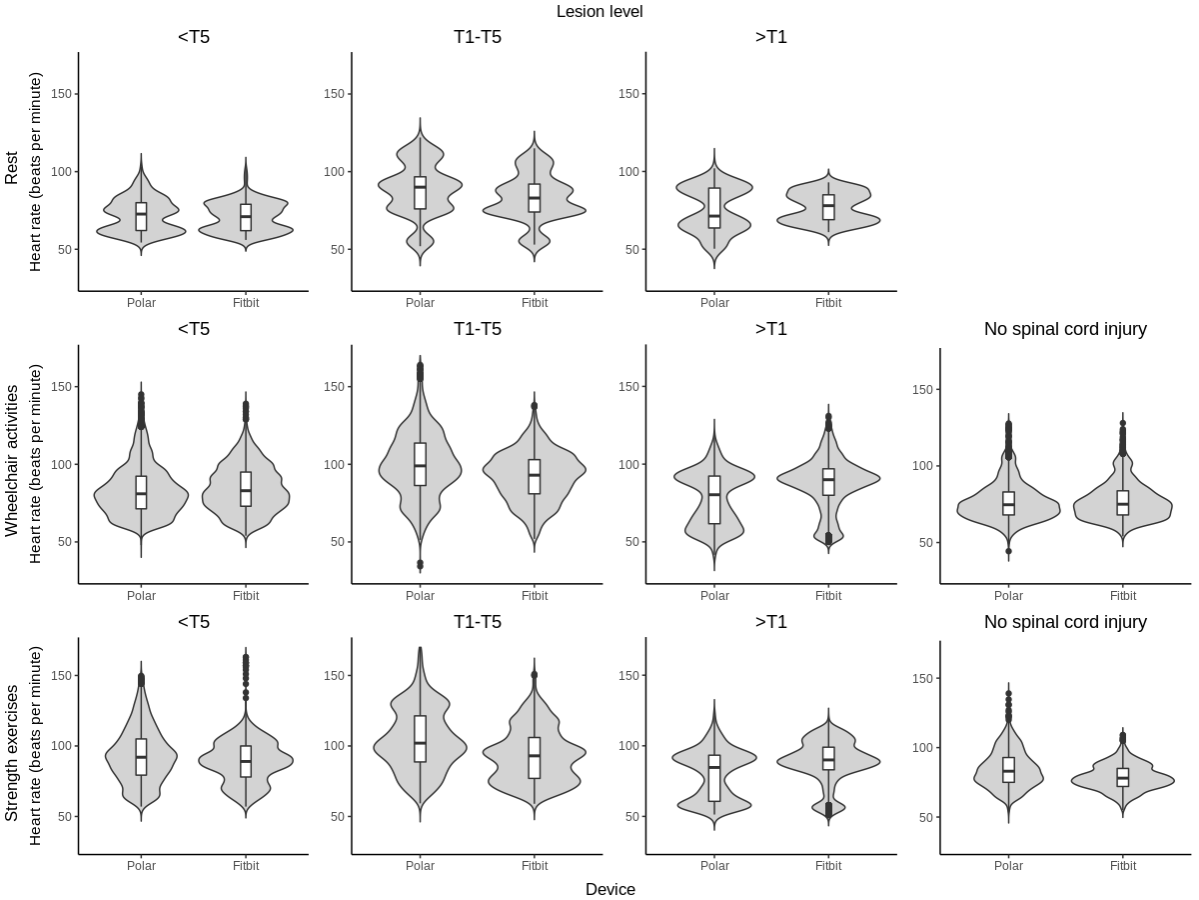
Violin plots of heart rate observations for Polar H7 and Fitbit Charge 2 divided by intensity from top to bottom in rest, wheelchair activities and strength exercise and divided by lesion level from left to right in lesion <T5, T1-T5, >T1, no spinal cord injury. Mean heart rate in beats per minute and IQRs are shown together with the distributions. T1: first thoracic vertebrae; T5: fifth thoracic vertebrae.

Table 1 shows the demographic characteristics of the 77% (34/44) wheelchair users with SCI and 23% (10/44) participants without SCI included in the analyses. Table 2 shows the descriptive statistics for the 21,732 HR samples measured by the Polar H7 HR monitor and the Fitbit Charge 2. These samples were taken during rest (1168 HR samples over a 5-minute period), wheelchair activities (12,016 HR samples), and strength exercises (8548 HR samples). In addition, the distributions in the HR samples are displayed visually in the violin plots shown in Figure 1. The violin plot displays the mirrored density plot in addition to the box plot, which displays summary statistics, such as the median and IQR. As shown in Table 2, the range of the HR samples from Polar H7 was wider than the HR estimates produced by the Fitbit Charge 2. The differences in the range of HRs became more pronounced when the lesion was above T5. However, further investigation showed that the range produced by the Polar H7 and Fitbit Charge 2 was quite similar for people with SCI above T1.

**Table 1:** Demographic characteristics of participants (N=44).

Characteristics		Lesion level					
		All lesions (n=34)	Below T5^a^ (n=16)	T5 and above (n=18)	T1^b^-T5 (n=10)	Above T1 (n=8)	No SCI^c^ (n=10)
**Gender**							
	Female	9	5	4	2	2	3
	Male	25	11	14	8	6	7
Age (years), mean (SD)		48.9 (12)	49.3 (13.7)	48.4 (10.9)	50.0 (9.9)	46.5 (12.5)	50.8 (10.1)
AIS (A/B/C/D)^d^		14/3/2/15	6/0/1/9	8/3/1/6	8/0/0/2	0/3/1/4	N/A^e^
Time since injury (years), mean (SD)		14.7 (11.6)	15.9 (13.3)	13.6 (10.1)	15.0 (11.7)	11.9 (8.1)	N/A
BMI, mean (SD)		24.2 (4.1)	23.5 (4)	24.7 (4.3)	25.7 (4.8)	23.6 (3.5)	26.0 (3.4)

^a^T5: fifth thoracic vertebrae.

^b^T1: first thoracic vertebrae.

^c^SCI: spinal cord injury.

^d^AIS: American Spinal Cord Injury Association Impairment Scale score.

^e^N/A: not applicable.

**Table 2:** Descriptive statistics of heart rate (HR) samples per activity, device, and lesion level.

Lesion level			HR samples		Polar H7 HR monitor, mean HR (SD; range)		Fitbit Charge 2, mean HR (SD; range)
**All activities**							
	All included	21,732		85.7 (19.7; 34.3-169.7)		85.6 (15.7; 50-163)	
	All lesions	17,211		89 (20.7; 34.3-169.7)		87.6 (16.2; 50-163)	
	<T5^a^	8172		86.3 (18.1; 48-149.7)		85.2 (15.3; 54-163)	
	>T5	9039		91.4 (22.4; 34.3-169.7)		89.9 (16.6; 50-151)	
	T1^b^-T5	5324		100.5 (21.2; 34.3-169.7)		91.8 (16.9; 52-151)	
	>T1	3715		78.3 (17.1; 41.7-121.7)		87.1 (15.7; 50-131)	
	No SCI^c^	4521		80.5 (13.3; 44.3-139)		77.8 (10.7; 54-128)	
**Rest**							
	All lesions	1168		78.2 (15.5; 50.3-122)		76.2 (12.7; 53-115)	
	<T5	538		72 (10.7; 54.3-103.3)		70.7 (9.3; 56-102)	
	>T5	630		83.6 (17; 50.3-122)		80.8 (13.4; 53-115)	
	T1-T5	397		88.3 (16.8; 52-122)		83 (14.8; 53-115)	
	>T1	233		75.6 (14; 50.3-102)		77 (9.3; 61-93)	
**Wheelchair activities**							
	All included	12,016		85.3 (19.2; 34.3-164)		85.4 (15.8; 50-139)	
	All lesions	9654		87.4 (20; 34.3-164)		87.5 (15.9; 50-139)	
	<T5	4434		83.1 (16.1; 48-145)		84.3 (15; 54-139)	
	>T5	5220		91 (22.2; 34.3-164)		90.2 (16.2; 50-138)	
	T1-T5	3119		99.8 (20.7; 34.3-164)		92.1 (15.8; 52-138)	
	>T1	2101		77.8 (17.3; 41.7-118.7)		87.3 (16.3; 50-131)	
	No SCI	2362		76.9 (12.5; 44.3-127.7)		77 (12; 54-128)	
**Strength exercises**							
	All included	8548		91.1 (20; 51.3-169.7)		87.1 (15.5; 51-163)	
	All lesions	6389		93.4 (21.4; 51.3-169.7)		90.0 (16.2; 51-163)	
	<T5	3200		93.6 (23.4; 51.3-169.7)		88.8 (14.9; 57-163)	
	>T5	3189		93.1 (19.2; 57-149.7)		91.2 (17.3; 51-151)	
	T1-T5	1808		104.4 (21.8; 59.3-169.7)		93.2 (18.6; 59-151)	
	>T1	1381		79.6 (17.3; 51.3-121.7)		88.5 (15; 51-119)	
	No SCI	2159		84.4 (13.1; 53.3-139)		78.6 (9.1; 55-109)	

^a^T5: fifth thoracic vertebrae.

^b^T1: first thoracic vertebrae.

^c^SCI: spinal cord injury.

### Mean Absolute Error

Overall, the Fitbit Charge 2 had a mean percentage error rate of 12.99% for people with SCI (Table 3), which is too high considering the standard acceptable MAPE is –10% to +10%. The MAPE of people with a lesion below T5 and between T1 and T5 was comparable with 11.16% and 10.16%, respectively, but for people with a lesion above T1, the MAPE was considerably higher (20.43%). People without SCI showed slightly better MAPE (8.09%) compared with people with lesions below T5 and between T1 and T5, as the MAPE was within the standard acceptable range of –10% to +10%. The MAPE was dependent on the type of activity performed by people with SCI. For rest, the overall MAPE was 6.5%, whereas the MAPE increased with the intensity of the activity to 12.97% for wheelchair activities and 14.2% for strength exercises. A similar trend was found in people without SCI, where the MAPE for strength exercise (8.39%) was slightly higher than the MAPE for wheelchair activities (7.82%). For each activity, a pattern exists where the MAPE increased with higher lesion levels. Taken together, the MAPE of the Fitbit Charge 2 only seemed within the acceptable range for people with SCI during rest. With higher lesion levels, Fitbit Charge 2 HR measurements were more off relative to the Polar H7 HR estimates.

**Table 3:** Device error statistics.

Lesion level		Heart rate samples	Error Fitbit Charge 2			Bland-Altman analysis	
			MAE^a^	MAPE^b^ (%)	ME^c^ (SD)	Lower LoA^d^	Upper LoA
**All activities**							
	All included	21,732	10.2	11.97	−1.6 (16)	−32.9	29.7
	All lesions	17,211	11.1	12.99	−1.3 (17.0)	−34.7	32.1
	<T5^e^	8172	9.6	11.16	−1.1 (14.7)	−30	27.8
	>T5	9039	12.4	14.64	−1.5 (18.9)	−38.5	35.5
	T1^f^-T5	5324	11.6	10.6	−8.7 (15.7)	−39.3	22
	>T1	3715	13.7	20.43	8.8 (18.4)	−27.3	44.8
	No SCI^g^	4521	7	8.09	−2.7 (10.8)	−23.9	18.5
**Rest**							
	All lesions	1168	5.2	6.5	−2.1 (9.0)	−19.7	15.6
	<T5	538	4.1	5.41	−1.2 (8.3)	−17.4	14.9
	>T5	630	6.2	7.43	−2.7 (9.5)	−21.4	15.9
	T1-T5	397	6.2	6.27	−5.2 (9.2)	−23.3	12.8
	>T1	233	6.2	9.39	1.5 (8.6)	−15.3	18.3
**Wheelchair activities**							
	All included	12,016	9.9	11.96	0.1 (15.4)	−30.1	30.3
	All lesions	9654	10.7	12.97	0.1 (16.4)	−32.1	32.3
	<T5	4434	9.2	11.29	1.2 (14.3)	−26.8	29.1
	>T5	5220	12	14.4	−0.8 (18)	−36.1	34.6
	T1-T5	3119	10	10.33	−7.7 (14.2)	−35.6	20.2
	>T1	2101	13.5	20.43	9.5 (18.2)	−26.3	45.2
	No SCI	2362	6.3	7.82	0.1 (10.2)	−19.9	20.2
**Strength exercises**							
	All included	8548	11.5	12.73	−4 (17.1)	−37.5	29.5
	All lesions	6389	12.7	14.2	−3.4 (18.8)	−40.1	33.4
	<T5	3200	11.1	11.94	−4.3 (15.6)	−34.9	26.3
	>T5	3189	14.4	16.47	−2.5 (21.4)	−44.4	39.5
	T1-T5	1808	13.8	12.03	−11.2 (18.5)	−47.5	25.2
	>T1	1381	15.1	22.29	8.9 (19.5)	−29.3	47.1
	No SCI	2159	7.7	8.39	−5.8 (10.6)	−26.6	14.9

^a^MAE: mean absolute error.

^b^MAPE: mean absolute percent error.

^c^ME: mean error.

^d^LoA: limits of agreement.

^e^T5: fifth thoracic vertebrae.

^f^T1: first thoracic vertebrae.

^g^SCI: spinal cord injury.

### Bland-Altman Analysis and 95% LoA

Table 3 shows the results from the Bland-Altman analysis, and Multimedia Appendix 1 shows the Bland-Altman plots. Across all lesion levels and activities, the mean error of the Fitbit Charge 2 was −1.3 (SD 17) bpm (lower LoA-upper LoA: −34.7 to 32.1 bpm) and MAE was 11.1. People without SCI showed a slightly larger mean error of −2.7 (SD 10.8) bpm (lower LoA-upper LoA: −23.9 to 18.5 bpm) but a smaller MAE of 7. Less agreement was observed in the group with a higher lesion level—mean error −1.1 (SD 14.7) bpm for the group with SCI lesions below T5 (lower LoA-upper LoA: −30 to 27.8 bpm), mean error −8.7 (SD 15.7) bpm (lower LoA-upper LoA: −39.3 to 22 bpm) for the group with SCI lesions between T1 and T5, and mean error 8.8 (SD 18.4) bpm (lower LoA-upper LoA: −27.3 to 44.8 bpm) for those with SCI lesions above T1. Although there were some outliers, Fitbit Charge 2 did not seem to systematically overestimate or underestimate HR values during rest in people with SCI. For the group with SCI lesions below T5, all outliers shown in Bland-Altman plots in Multimedia Appendix 1 during all 3 activities were from 3 separate participants. During rest, the overall mean error for people with SCI was −2.1 (SD 9) bpm (lower LoA-upper LoA: −19.7 to 15.6 bpm). Here, the agreement seemed lowest for the group with an SCI between T1-T5 with a mean error of −5.2 (SD 9.2) bpm (lower LoA-upper LoA: −23.3 to 12.8 bpm) compared with a mean error of −1.2 (SD 8.3) bpm (lower LoA-upper LoA: −17.4 to 14.9 bpm) for those with an SCI below T5 and a mean error of 1.5 (SD 8.6) bpm (lower LoA-upper LoA: −15.3 to 18.2 bpm) for those with an SCI above T1. In contrast, investigation of the plots presented in Multimedia Appendix 1 showed that during wheelchair activities and strength exercises, a trend toward overestimation for values below 100 bpm and an underestimation for observations with higher bpm was present. These trends seemed more pronounced during the strength exercises where the mean error was −3.4 (SD 18.8) bpm (lower LoA-upper LoA: −40.1 to 33.4 bpm) compared with an overall mean error of 0.1 (SD 16.4) bpm (lower LoA-upper LoA: −32.1 to 32.3 bpm) during wheelchair activities. A similar trend was found during strength exercise in those without an SCI, with a mean error of −5.8 (SD 10.6) bpm (lower LoA-upper LoA: −26.6 to 14.9 bpm) for strength exercise compared with a mean error of 0.1 (SD 10.2) bpm (lower LoA-upper LoA: −19.9 to 20.2 bpm) for wheelchair activities. Overall, Bland-Altman plots showed a trend toward overestimation of HR values for observations between 80 and 100 bpm in people with SCI lesions below T5. This was, to a lesser extent, also observed in general for people with SCI lesions above T1. In contrast, the Fitbit Charge 2 mostly underestimated the HR values of observations with ≥80 bpm in people with SCI between T1-T5.

### Concordance Class Correlation

Overall, across all activities and all included groups, the Fitbit Charge 2 had a moderate agreement with the Polar H7 HR monitor (CCC=0.596, 95% CI 0.587-0.604). During rest, this agreement was stronger (CCC=0.791, 95% CI 0.770-0.810) and as intensity increased, this agreement became weaker; during wheelchair activities CCC_activities_=0.615 (95% CI 0.605-0.626) and during strength exercises CCC_strength_=0.531 (95% CI 0.517-0.545). Overall, the agreement was stronger for those with an SCI lower than T1 or no SCI and became much weaker for the group with SCI above T1: CCC_noSCI_=0.585 (95% CI 0.567-0.603), CCC_<T5_=0.613 (95% CI 0.599-0.626), CCC_T1−T5_=0.605 (95% CI 0.590-0.620), and CCC_>T1_=0.328 (95% CI 0.302-0.353). Agreement was weak for people with a lesion above T1 during wheelchair activities (CCC_>T1activities_=0.354, 95% CI 0.321-0.386) and strength exercises (CCC_>T1strength_=0.238, 95% CI 0.195-0.281). For lesions between T1 and T5 and lesions below T5, the agreement was moderate. Moderate (CCC_no SCI activities_=0.653, 95% CI 0.629-0.675) to low (CCC_no SCIstrength_=0.490, 95% CI 0.464-0.516) agreements were found for those without SCI, as shown in Table 4.

**Table 4:** Concordance class correlation based on lesion groups and activities.

Lesion level		Heart rate samples	Concordance class correlation (95% CI)
**All activities**			
	All included	21,732	0.596 (0.587-0.604)
	All lesions	17,211	0.577 (0.567-0.586)
	<T5^a^	8172	0.613 (0.599-0.626)
	>T5	9039	0.541 (0.527-0.554)
	T1^b^-T5	5324	0.605 (0.590-0.620)
	>T1	3715	0.328 (0.302-0.353)
	No SCI^c^	4521	0.585 (0.567-0.603)
**Rest**			
	All lesions	1168	0.791 (0.770-0.810)
	<T5	538	0.659 (0.609-0.703)
	>T5	630	0.792 (0.764-0.817)
	T1-T5	397	0.788 (0.751-0.820)
	>T1	233	0.736 (0.684-0.780)
**Wheelchair activities**			
	All included	12,016	0.615 (0.605-0.626)
	All lesions	9654	0.586 (0.573-0.599)
	<T5	4434	0.577 (0.558-0.597)
	>T5	5220	0.567 (0.550-0.584)
	T1-T5	3119	0.645 (0.627-0.663)
	>T1	2101	0.354 (0.32-0.386)
	No SCI	2362	0.653 (0.629-0.675)
**Strength exercises**			
	All included	8548	0.531 (0.517-0.545)
	All lesions	6389	0.503 (0.486-0.520)
	<T5	3200	0.567 (0.545-0.534)
	>T5	3189	0.457 (0.431-0.482)
	T1-T5	1808	0.505 (0.475-0.534)
	>T1	1381	0.238 (0.195-0.281)
	No SCI	2159	0.490 (0.464-0.516)

^a^T5: fifth thoracic vertebrae.

^b^T1: first thoracic vertebrae.

^c^SCI: spinal cord injury.

## Discussion

### Principal Findings

This is, to our knowledge, the first study to assess the HR accuracy of Fitbit Charge 2 in people with SCI, or more specifically, to assess the effects of lesion level on PPG-based HR accuracy. With an overall MAPE of 12.99% for the Fitbit Charge 2, the standard acceptable error of –10% to +10% was not met, and the outcomes were worse than in earlier research in able-bodied populations [17,20]. As the intensity of the activity increased, the HR accuracy of Fitbit Charge 2 worsened, which is in line with previous research [18-20]. Moreover, there seems to be a clear effect of lesion level, as the highest lesion group (>T1) showed drastically lower accuracy on Fitbit HR recordings on all intensities, compared with lower lesion level groups. This could possibly contribute to a more severely affected sympathetic innervation.

Compared with previous research in able-bodied individuals, our findings showed poorer outcomes for both MAPE and agreement rate during wheelchair activities and strength exercises. Previous research on the accuracy of HR measurements of the Fitbit Charge 2 that included similar activities (seated rest, activities of daily living, strength exercises) showed a MAPE range of 5.93% to 9.88% in able-bodied individuals. A similar range was found in this study in people without SCI (7.82%-8.39%) [17,20]. In all people with SCI, the MAPE range varied between 6.5% and 14.2%. During seated rest, our findings showed a stronger association (CCC=0.791) between the Fitbit Charge 2 and Polar H7 HR monitor compared with a moderate association in previous research (CCC=0.561) [17]; however, agreement and error in all other activities showed poorer results and worsened as intensity increased in people with SCI. The reduced accuracy with increasing intensities is in line with the literature [18,19], but accuracy worsened more in people with SCI during wheelchair activities (CCC=0.586; MAPE 12.97%) and strength exercises (CCC=0.503; MAPE 14.2%) than in people without SCI during wheelchair activities (CCC=0.653; MAPE 7.82%) and strength exercises (CCC=0.490; MAPE 8.39%) and previous literature (activities of daily living: CCC=0.739; MAPE 8.29%; strength exercise: CCC=0.72; MAPE 9.8%; [17,20]). It could be argued that performing activities in a wheelchair could influence the agreement of HR recording in wrist-worn wearables in general as the CCC values in this study tend to be lower, even in people without SCI. To perform certain activities in a wheelchair, the wrist is often repetitively pressed and bumped against the rim of the wheel during propulsion, which could continuously affect the PPG connection as the pressure between the sensor and skin fluctuates [39]. This could, at least in part, explain the overall poorer accuracy of the Fitbit Charge 2 during wheelchair activities in people with and without SCI in this study compared with previous findings in able-bodied individuals. However, this would not explain the drastically decreased HR accuracy of the Fitbit Charge 2 in the higher lesion level (>T1) group. Therefore, it is very likely that a more severely imbalanced ANS negatively affects the accuracy [26].

It is remarkable that the T1-T5 group showed no clear difference from the <T5 group, as the sympathetic pathway is affected at lesion levels above T6 and an imbalance between the sympathetic and parasympathetic system is most likely present, which controls HR and blood pressure [24]. As there is a major difference between Polar H7 and Fitbit Charge 2 in the technique used to measure the obtained HR outcomes, it seems likely that this difference causes a drop in accuracy and agreement during the Fitbit Charge 2 HR recording. Because Fitbit Charge 2 HR recording is based on blood pressure differences, and autonomic control of the blood vessels in the upper body is controlled between segments T1 and T4, it was expected to observe differences in the T1-T5 group as well as in the >T1 group compared with the <T5 group. However, it appears that as long as there is some innervation left and not all sympathetic innervation of the blood vessels is affected, HR accuracy measured by PPG is only slightly reduced. The accuracy only seems to drop at lesion levels above T1, as there is possibly no sympathetic innervation left of the blood vessels in the lower parts of the upper limbs [40]. In addition, people with tetraplegia are more likely to show lower blood pressure compared with people with paraplegia or able-bodied individuals caused by reduced sympathetic activity [41]. Therefore, hypotension is a common phenomenon among people with tetraplegia, which could possibly influence the accuracy of PPG-based HR recordings as it deviates from the regular expected signal [42,43].

The severity of reduced sympathetic innervation is not necessarily related to neurological lesion completeness, which is often expressed using the American Spinal Cord Injury Association Impairment Scale score. This scale is based on the presence of motor or sensory function, where a complete injury is defined as the absence of both motor and sensory function below the lesion, and an incomplete lesion is defined as any reduced presence of motor or sensory function below the lesion [44]. However, research has shown that this classification does not necessarily include autonomic function, because sympathetic activity has been detected in athletes with complete cervical SCI lesions [45]. Although lesion level clearly influences the ANS and, therefore, Fitbit Charge 2 HR accuracy, the effect of completeness of the lesion on motor, sensory, and autonomic function remains unknown. Therefore, future studies should test autonomic function separately from neurological lesions in people with SCI to gain better insight on the effect of autonomic function on HR accuracy based on PPG signals.

### Strengths and Limitations

A strength of this study was the relatively large sample size of people with SCI, in which the distribution among the different lesion level groups, which were based on physiological differences determined by the literature, was fairly even and the direct comparison between people with and without SCI [24,26,40]. Analyses were performed, when possible, according to the methodological approaches suggested by Nelson and Allen [17], van Lier et al [34], and Sartor et al [33]. Activities and exercises mimicked real-life situations, which increased the ecological validity. Participants with SCI performed the tasks in their own wheelchair, at their own speed in relatively short time bouts, representing real-life situations better than prolonged steady-state activities. A suitable wheelchair was provided to the participants without SCI. Outcomes were analyzed as a whole and divided by lesion group and rest, wheelchair activities, and strength exercises to gain insight on both the effect of intensity and lesion level on the accuracy.

However, there are some limitations to the design and analysis. The reference device used, a Polar H7 HR monitor, is not considered a gold standard. A 3-lead ECG HR monitor device would have served better as a reference device. However, the Polar H7 HR monitor shows a high correlation with a 3-lead ECG (Intraclass Correlation Coefficient=0.98) and is therefore a good alternative [30]. In addition, HR outcomes from both devices were provided without raw signals (raw ECG signals and interbeat intervals). Ideally, one would obtain all raw information as algorithms to convert raw signals into the reported HR are often confidential and unknown. Firmware versions were, therefore, reported to take into account any sealed changes in such algorithms and to allow for the replication of results. HR was collected at the highest possible sample rate for Fitbit Charge 2, as intraday time series access was provided by Fitbit for research purposes. As measurements were performed within a larger study on energy expenditure in people with SCI, the Polar H7 was connected to an indirect calorimetry device during measurements. The output provided by this device was given on a breath-by-breath basis, meaning the HR sample rate for the Polar H7 varied per minute and was determined by the breathing rate of the participant, which eventually provided a lower HR sample rate than preferred. The number of data points available for each activity to analyze reduced when the lesion level increased, as several participants were not able to perform certain wheelchair activities or strength exercises because of the severity of their impairment, present injuries, or risks. In addition, no information was collected on the environmental conditions or skin information that could possibly affect the PPG signal [33]. However, because all measurements were performed at the same location within the same rooms, temperature and light were similarly regulated during all the measurements. Unfortunately, no blood pressure data were collected during the measurement to strengthen our findings. Therefore, it is advisable to combine HR recordings together with continuous blood pressure data in future research to confirm our findings.

### Practical Implementations

HR data obtained with the PPG technique during activities, especially during high intensities in people with a high lesion level (>T1), could provide inaccurate HR data in people with SCI. Therefore, it is advised to avoid using PPG-based HR measurements for medical purposes in people with SCI with a cervical lesion level (>T1). However, despite a possible discrepancy in HR recordings, outcomes can still be of value in situations where the consequences of inaccurate HR data are low, for example, to get a global impression of energy expenditure and exercise intensity during physical activities in daily life.

### Conclusions

The overall accuracy of the Fitbit Charge 2 HR measurements in people with SCI did not reach the standard acceptable error of –10% to +10%. With increasing intensity, the HR accuracy of the Fitbit Charge 2 was further reduced in people with SCI compared with its HR accuracy in able-bodied individuals. In addition, HR accuracy is related to lesion level, where a high SCI lesion (>T1) negatively affects HR accuracy. Accuracy seems to worsen more in high lesion levels with increasing intensities. A clear reduction in accuracy was found in the lesion group >T1 during wheelchair activities and strength exercises. This suggests that PPG-based HR accuracy is affected in people with SCI, as blood pressure responses during activity are possibly altered because of an affected ANS. Therefore, PPG-based HR measurements during activities should be taken with caution in people with SCI, especially in those with cervical SCI lesions.

## Appendix

Multimedia Appendix 1 Bland-Altman plots.

## Abbreviations

ANS: autonomic nervous system
bpm: beats per minute
CCC: concordance correlation coefficient
ECG: electrocardiography
HR: heart rate
LoA: limits of agreement
MAE: mean absolute error
MAPE: mean absolute percentage error
PPG: photoplethysmography
SCI: spinal cord injury
T1: first thoracic vertebrae
T5: fifth thoracic vertebrae

## References

[1] van den Berg-Emons RJ, Bussmann JB, Stam HJ (2010). Accelerometry-based activity spectrum in persons with chronic physical conditions. Arch Phys Med Rehabil.

[2] Nevin AN, Steenson J, Vivanti A, Hickman IJ (2016). Investigation of measured and predicted resting energy needs in adults after spinal cord injury: a systematic review. Spinal Cord.

[3] Felleiter P, Krebs J, Haeberli Y, Schmid W, Tesini S, Perret C (2017). Post-traumatic changes in energy expenditure and body composition in patients with acute spinal cord injury. J Rehabil Med.

[4] Buchholz AC, McGillivray CF, Pencharz PB (2003). Differences in resting metabolic rate between paraplegic and able-bodied subjects are explained by differences in body composition. Am J Clin Nutr.

[5] Chun SM, Kim H, Shin HI (2017). Estimating the Basal metabolic rate from fat free mass in individuals with motor complete spinal cord injury. Spinal Cord.

[6] Weaver FM, Collins EG, Kurichi J, Miskevics S, Smith B, Rajan S, Gater D (2007). Prevalence of obesity and high blood pressure in veterans with spinal cord injuries and disorders: a retrospective review. Am J Phys Med Rehabil.

[7] Myers J, Lee M, Kiratli J (2007). Cardiovascular disease in spinal cord injury: an overview of prevalence, risk, evaluation, and management. Am J Phys Med Rehabil.

[8] Vincent J (2008). Understanding cardiac output. Crit Care.

[9] Gordan R, Gwathmey JK, Xie L (2015). Autonomic and endocrine control of cardiovascular function. World J Cardiol.

[10] Achten J, Jeukendrup AE (2003). Heart rate monitoring: applications and limitations. Sports Med.

[11] Allen J (2007). Photoplethysmography and its application in clinical physiological measurement. Physiol Meas.

[12] Sviridova N, Sakai K (2015). Human photoplethysmogram: new insight into chaotic characteristics. Chaos Solit Fract.

[13] Alnaeb M, Alobaid N, Seifalian A, Mikhailidis D, Hamilton G (2007). Optical techniques in the assessment of peripheral arterial disease. Curr Vasc Pharmacol.

[14] Zhang Y, Liu B, Zhang Z (2015). Combining ensemble empirical mode decomposition with spectrum subtraction technique for heart rate monitoring using wrist-type photoplethysmography. Biomed Sign Process Control.

[15] Castaneda D, Esparza A, Ghamari M, Soltanpur C, Nazeran H (2018). A review on wearable photoplethysmography sensors and their potential future applications in health care. Int J Biosens Bioelectron.

[16] de Zambotti M, Baker FC, Willoughby AR, Godino JG, Wing D, Patrick K, Colrain IM (2016). Measures of sleep and cardiac functioning during sleep using a multi-sensory commercially-available wristband in adolescents. Physiol Behav.

[17] Nelson BW, Allen NB (2019). Accuracy of consumer wearable heart rate measurement during an ecologically valid 24-hour period: intraindividual validation study. JMIR Mhealth Uhealth.

[18] Boudreaux B, Hebert E, Hollander D, Williams B, Cormier C, Naquin M, Gillan W, Gusew E, Kraemer R (2018). Validity of wearable activity monitors during cycling and resistance exercise. Med Sci Sports Exerc.

[19] Dooley EE, Golaszewski NM, Bartholomew JB (2017). Estimating accuracy at exercise intensities: a comparative study of self-monitoring heart rate and physical activity wearable devices. JMIR Mhealth Uhealth.

[20] Reddy RK, Pooni R, Zaharieva DP, Senf B, El Youssef J, Dassau E, Doyle Iii FJ, Clements MA, Rickels MR, Patton SR, Castle JR, Riddell MC, Jacobs PG (2018). Accuracy of wrist-worn activity monitors during common daily physical activities and types of structured exercise: evaluation study. JMIR Mhealth Uhealth.

[21] Wright SP, Hall BT, Collier SR, Sandberg K (2017). How consumer physical activity monitors could transform human physiology research. Am J Physiol Regul Integr Comp Physiol.

[22] Dias D, Cunha J (2018). Wearable health devices-vital sign monitoring, systems and technologies. Sensors (Basel).

[23] Perez MV, Mahaffey KW, Hedlin H, Rumsfeld JS, Garcia A, Ferris T, Balasubramanian V, Russo AM, Rajmane A, Cheung L, Hung G, Lee J, Kowey P, Talati N, Nag D, Gummidipundi SE, Beatty A, Hills MT, Desai S, Granger CB, Desai M, Turakhia MP, Apple Heart Study Investigators (2019). Large-scale assessment of a smartwatch to identify atrial fibrillation. N Engl J Med.

[24] Grigorean V, Sandu A, Popescu M, Iacobini M, Stoian R, Neascu C, Strambu V, Popa F (2009). Cardiac dysfunctions following spinal cord injury. J Med Life.

[25] Bent B, Goldstein BA, Kibbe WA, Dunn JP (2020). Investigating sources of inaccuracy in wearable optical heart rate sensors. NPJ Digit Med.

[26] Grimm DR, DeMeersman RE, Garofano RP, Spungen AM, Bauman WA (1995). Effect of provocative maneuvers on heart rate variability in subjects with quadriplegia. Am J Physiol-Heart Circul Physiol.

[27] (2002). Method comparison and bias estimation using patient samples; approved guideline - second edition. Clinical And Laboratory Standards Institute [CLSI].

[28] Teasell RW, Arnold JO, Krassioukov A, Delaney GA (2000). Cardiovascular consequences of loss of supraspinal control of the sympathetic nervous system after spinal cord injury. Arch Phys Med Rehabil.

[29] Sachdeva R, Nightingale TE, Krassioukov AV (2019). The blood pressure pendulum following spinal cord injury: implications for vascular cognitive impairment. Int J Mol Sci.

[30] Pasadyn SR, Soudan M, Gillinov M, Houghtaling P, Phelan D, Gillinov N, Bittel B, Desai MY (2019). Accuracy of commercially available heart rate monitors in athletes: a prospective study. Cardiovasc Diagn Ther.

[31] The Association for Advancement of Medical Instrumentation (1986). American National Standard. SMPTE J.

[32] Lehnert B (2015). BlandAltmanLeh: plots (slightly extended) Bland-Altman plots. Cran.R-Project.

[33] Sartor F, Papini G, Cox LG, Cleland J (2018). Methodological shortcomings of wrist-worn heart rate monitors validations. J Med Internet Res.

[34] van Lier HG, Pieterse ME, Garde A, Postel MG, de Haan HA, Vollenbroek-Hutten MM, Schraagen JM, Noordzij ML (2020). A standardized validity assessment protocol for physiological signals from wearable technology: methodological underpinnings and an application to the E4 biosensor. Behav Res Methods.

[35] Zaki R, Bulgiba A, Nordin N, Azina Ismail N (2013). A systematic review of statistical methods used to test for reliability of medical instruments measuring continuous variables. Iran J Basic Med Sci.

[36] Altman DG, Bland JM (1983). Measurement in medicine: the analysis of method comparison studies. Statistician.

[37] Lin LI (1989). A concordance correlation coefficient to evaluate reproducibility. Biometrics.

[38] Signorell A (2020). DescTools: tools for descriptive statistics. Cran.R-Project.

[39] Teng XF, Zhang YT (2004). The effect of contacting force on photoplethysmographic signals. Physiol Meas.

[40] West C, Alyahya A, Laher I, Krassioukov A (2013). Peripheral vascular function in spinal cord injury: a systematic review. Spinal Cord.

[41] Mathias C, Frankel H, Frankel H, Bruyn G, Klawans H, Vinken P (1992). The cardiovascular system in tetraplegia and paraplegia. Spinal Cord Trauma: Handbook of Clinical Neurology.

[42] Lehmann K, Lane J, Piepmeier J, Batsford W (1987). Cardiovascular abnormalities accompanying acute spinal cord injury in humans: incidence, time course and severity. J Am Coll Cardiol.

[43] Lemay M, Bertschi M, Sola J, Renevey P, Parak J, Korhonen I (2015). Chapter 2.3 - Application of optical heart rate monitoring. Wearable Sensors - Fundamentals, Implementation and Applications.

[44] Kirshblum SC, Burns SP, Biering-Sorensen F, Donovan W, Graves DE, Jha A, Johansen M, Jones L, Krassioukov A, Mulcahey M, Schmidt-Read M, Waring W (2013). International standards for neurological classification of spinal cord injury (Revised 2011). J Spinal Cord Med.

[45] West CR, Romer LM, Krassioukov A (2013). Autonomic function and exercise performance in elite athletes with cervical spinal cord injury. Med Sci Sports Exerc.

