# Supplementary figures and images for: Accuracy of Heart Rate Measurement by the Fitbit Charge 2 During Wheelchair Activities in People With Spinal Cord Injury: Instrument Validation Study
Source: JMIR Rehabil Assist Technol. 2022 Jan 19;9(1):e27637. doi: 10.2196/27637 (PMC8811691; doi:10.2196/27637)

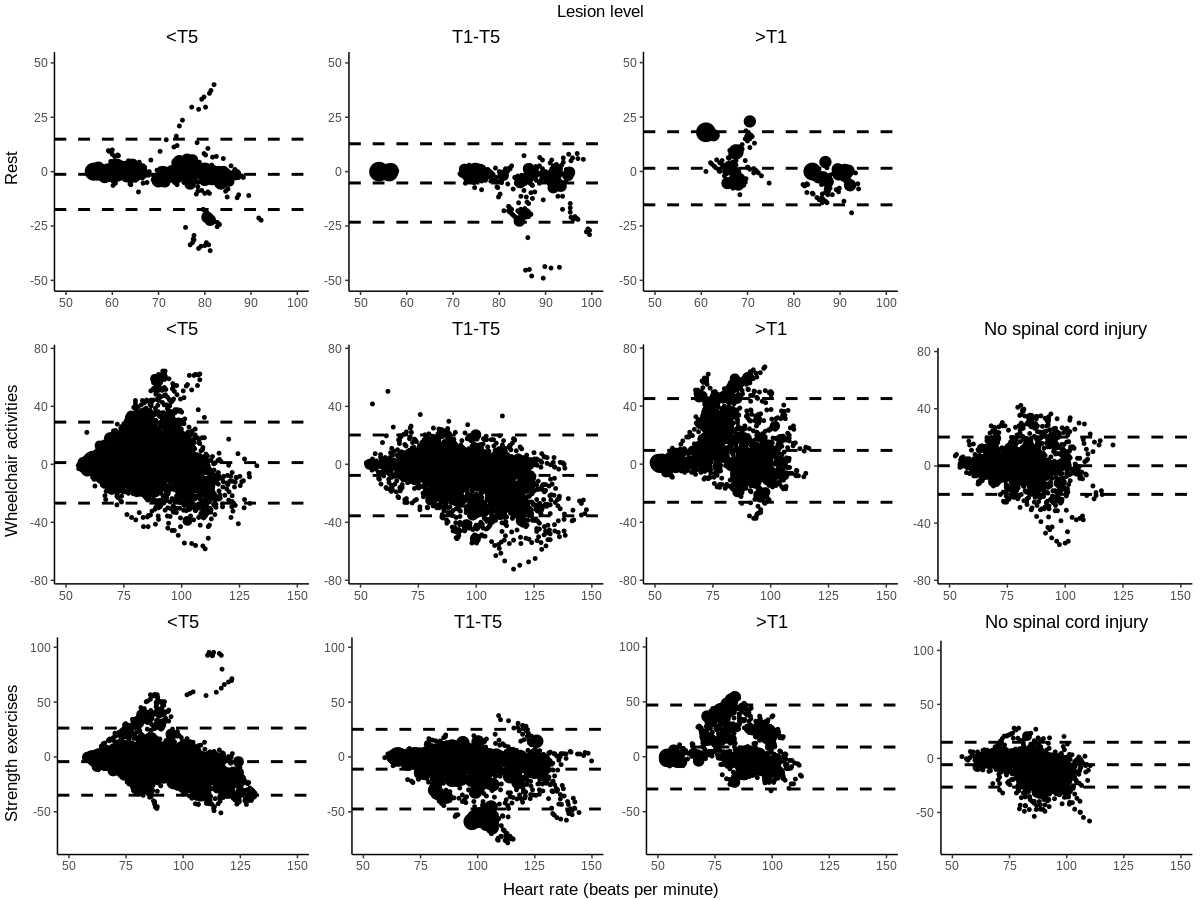

Supplement: Multimedia Appendix 1 [file rehab_v9i1e27637_app1.png]
